# Case of Fistulous Abscess of the Liver Communicating with the Gallbladder; Dilatation and Cauterisation of the Fistulous Canal; Extraction of Sixteen Biliary Calculi; Cure

**Published:** 1846-08

**Authors:** 


*Case of Fistulous Abscess of the Liver, communicating with the gall-bladder ; dilatation and cauterisation of the fistulous canal ; extraction of sixteen biliary calculi ; cure.* By Doctor Levacher.—Cases of abscess of the liver opening externally and giving passage to biliary calculi are not very frequent in medicine ; Boyer notices one related by Borrichius, in which there were more than four hundred calculi passed. Breschet in his article Calculus in the 'Dictionnaire de Medecine' gives two others reported by Thilésius and Stalpart Van-der Wiel; Thilésius says that in a case of abscess of the liver there came away in the course of nine years, five or six hundred small stones ; and Stalpart Van-der-Wiel mentions the passage in this way of one calculus of the size of a pigeon's egg. In speaking on this subject I would refer the reader to the excellent memoir of J. L. Petit on tumors of the gall-bladder and abscesses of the kind I have mentioned. He has given rules for their treatment, and has established them by a large number of cases. It is always much to be regretted that the reports are deficient in necessary details ; that which I am about to relate, besides being very full and complete, will be found interesting I hope both from the description of the treatment made use of and from the perfect and continued cure.

In April 1838 I was called to attend a lady, Mrs. J. —— from Tulle in the department of Correze, residing in the rue de Boucher, No. 3.

She was 23 years of age, of a light florid complexion, rather tall, and quite fleshy, and appeared at first sight to enjoy perfect health. She told me that three years before, in consequence of a violent blow on the right side, she had experienced at first a continued pain accompanied with chills and fever; this was followed by stitches, and at last, after suffering for more than a month, an abscess formed, which opened spontaneously, and was pronounced by the physician of the place to be an abscess of the liver. The cure of this abscess had been very tedious, and a purulent exudation lasted for more than two months. I could learn nothing from the patient about the character of the pus. For the last two or three months she had experienced anew the lancinating pain in the right side accompanied with a slight attack of fever;—in consequence of this relapse, which was much less painful than the primitive accident, she noticed a small abscess below the cicatrice of the former one; this soon opened, and a clear pus wept from it; and being very much annoyed by her condition, she left Tulle and came to Paris to obtain if possible a cure.

On placing the patient in bed on her back, I found her pelvis and abdomen both of unusual size; the cellular tissue was likewise well developed. Near the linea alba on the right side, and three fingers breadth below the umbilicus, was a fistulous orifice. The deep star-shaped cicatrice of the old abscess was to be seen at about two fingers breadth above and to the right of the umbilicus; this cicatrice seemed to show, by its distance from the liver, that the old abscess had spread of itself and opened externally by a canal which had become fistulous.

The quantity of fat in the abdominal walls did not allow a close examination of the liver, but to the touch it appeared to be somewhat hypertrophied; palpation and percussion produced no acute pain but an unpleasant feeling; at the two superior thirds of the organ, the sounds were mat, but above that they were natural; the false ribs could be easily detached by pressure, and there was evidently no adhesion with the diaphragm.

Her bowels were regularly moved and the stools natural; the urine was in good condition; there was no fever, and all the functions were well carried on.

A sero-purulent discharge took place on pressure over the fistulous canal. Into this canal I passed a grooved director, which followed it without any difficulty in a transverse direction for about four inches and then suddenly stopped: here there was evidently a sudden turn in the canal, as we shall see hereafter. I divided the tissues immediately on the director up to the point where it was stopped; a suitable dressing was then applied. After some days I found that the canal still existed; I passed the director again, and now it went from below upwards by the side of the old cicatrice, and after some difficulties at this point it continued a little further. I laid open the canal again up to this last point; and here some sinuosities which interfered with the passage of the director were destroyed. The instrument then passed freely for one or two inches in the direction of the superior part of the liver towards the epigastrium and there stopped. The extremity of the director was free, and could be moved in various directions, on doing which a very distinct crepitation could be perceived, and it seemed as though it was touching a chalky substance, rather than a resisting calculus.

On account of this fact, which seemed to me a serious one, I desired a consultation and called in one of our clinical professors of surgery. The professor examined the patient, sounded the fistula, and thought there was no affection of the liver, but that the director passed into the interior of a deep old abscess, whose position could not be positively determined; he thought that the crepitation which was felt with the director came from old calcareous deposits in the sac, as is sometimes the case. He agreed with me that the fistula should be kept open and gradually dilated by the aid of sponges properly prepared. His prognosis was more unfavourable than mine, because I thought a cure very possible. He was of opinion that notwithstanding the excellent health of the patient, serious accidents would soon come on, and told me, and the husband also, that he considered the case to be necessarily a fatal one sooner or later.

I asked of the husband a new consultation of several surgeons, either together or separately, but my request was denied. They would not hear of a consultation, and the patient was put wholly under my care, with the assurance, that whatever might be the result, they would be thankful for my attentions.

I placed the patient under a treatment both general and local. I advised perfect rest in bed, and put her on a low diet, such as is suitable in affections of the liver. She took, as occasion required, whole or partial tepid baths, enemata, &c. Perfect freedom of the bowels was kept up by gentle laxatives, and her drinks were diluent and cooling.

I commenced a gradual dilatation of the canal by means of sponges, prepared by wetting them with a solution of gum and then compressing them until dry. I succeeded with some difficulty, by means of the sponges and cauterizations, in enlarging the whole canal to the size of my little finger, but notwithstanding this the sponges were always firmly grasped, especially by that part of the canal corresponding with the entrance into the peritoneum; at this point there was a sort of neck on which the sponges had but little effect; they were repeatedly torn, and it was with great difficulty that I removed the fragments, which was done by means of small straight forceps.

With this degree of dilatation I could distinctly perceive the existence of calculi by means of a sound; these could only be distinguished to the right and left, no sound being given immediately at the bottom of the opening. I had arrived at this stage of the treatment, when, on removing the sponge one day, a blackish calculus with facettes, and of the size of a pea, escaped, then came a gush of green bile, and again two other calculi of the same size.

I introduced the sound and still found nothing immediately before the instrument, but to the right and to the left there existed, very evidently, a calculous body, resisting, and of a much greater size than those which had been passed. It was impossible to determine whether there were two bodies or one, even when I made use of the female catheter, which is curved at its extremity.

The general health of the patient continued good ; all the functions were well carried on ; her sleep was excellent, and she merely suffered a slight pain in the region of the upper part of the liver.

Finding that it was necessary to increase the dilatation to double the size I had already obtained, I had recourse to catgut strings. I had some prepared of the size of the little finger, of the ring finger and of the index ; these I expected would become one-third larger by remaining in the fistulous canal for twelve hours, where they would absorb the moisture of the parts. This method of treatment answered very well, and at each dressing a small quantity of bile escaped, and now and then a small calculus. At the end of five or six days the dilatation was equal in size to the thumb, and fourteen calculi, all of the same size, had been passed.

I had kept up the dilatation to the same extent for some days without any more calculi being passed, when one day, on passing the sound, I found a large calculus engaged in the commencement of the canal. I increased the dilatation somewhat so as to facilitate its passage, and the next day but one it had advanced two-thirds of the distance—on the fourth day it was retained by nothing but the neck caused by the peritoneum, which still existed. I now undertook its removal with a pair of straight polypus forceps: by carefully introducing them, first shut, then gradually opened, I succeeded in seizing the calculus, and tried to extract it, but the resistance of the neck prevented me until it broke into three or four pieces. I then carefully removed these with a pair of small forceps. On reuniting the fragments I formed a calculus of about the size and form of a pigeon's egg, and what is very remarkable, it had no facettes, neither was its color, which was of a brownish yellow, the same as that of the small calculi : its internal structure, however, was the same. It was composed of crystallised biliary adipocire, known under the name of cholesterine. The sound, on being introduced, gave no signs of calculus on the left side, but there still existed the same sensations on the right.

Three or four days after, another calculus presented itself and came away just as the former; it was broken into several portions, which, when put together, were of the same size and form as the preceding.

From the moment these two calculi were passed, the patient experienced perfect relief. I kept up the same degree of dilatation for a week longer, but not finding during this time any calculus with the sound, I began to use smaller cords. In a few days the canal had diminished to the size of a large goose-quill ; there was no more passage of bile, the patient's appetite and strength increased, her *embonpoint*, which she had lost somewhat, returned, and she wished to get up, and thought she could easily take a walk.

I thought it advisable to keep up some degree of dilatation two weeks longer, and several times cauterised the canal with a piece of catgut slightly soaked in a solution of lunar caustic. I allowed my patient to leave her bed and lie on a sofa during the day, and at the end of the two weeks finding no signs of calculus with the sound, I gradually lessened the dilatation.

During this time I applied frictions over the region of the liver with mercurial ointment, and afterwards covered the part with mild poultices. I recommended to the patient the use of the *eau de Vichy* internally, and of flannel for covering. I soon left the wound to itself, and in a few days cicatrization was completed. Three months had now elapsed since the patient was placed under my charge.

This case occurred, as I have before mentioned, in the year 1838, last year, 1844, I had the pleasure of seeing Mrs. J——, who lives now in Paris; her cure is perfect. Since her treatment she has enjoyed uninterrupted health, and has never suffered the east colic, or anything indicating a calculus affection of the liver.—*Journal de Chirugie.*

